# Post-Surgical Prognosis of Patients with Pineoblastoma: A Systematic Review and Individual Patient Data Analysis with Trends over Time

**DOI:** 10.3390/cancers15133374

**Published:** 2023-06-27

**Authors:** Khizar R. Nandoliya, Nishanth S. Sadagopan, Vineeth Thirunavu, Ethan J. Houskamp, Constantine L. Karras, Rahul K. Chaliparambil, Nikhil Sriram, Pouya Jamshidi, David R. Raleigh, Rimas V. Lukas, Stephen T. Magill

**Affiliations:** 1 Department of Neurological Surgery, Malnati Brain Tumor Institute, Northwestern University, Chicago, IL 60611, USA; khizar.nandoliya@northwestern.edu (K.R.N.); constantine.karras@northwestern.edu (C.L.K.);; 2 Department of Pathology, Northwestern University, Chicago, IL 60611, USA; 3 Department of Radiation Oncology, University of California San Francisco, San Francisco, CA 94143, USA; 4 Department of Neurological Surgery, University of California San Francisco, San Francisco, CA 94143, USA; 5 Department of Pathology, University of California San Francisco, San Francisco, CA 94143, USA; 6 Department of Neurology, Malnati Brain Tumor Institute, Northwestern University, Chicago, IL 60611, USA

**Keywords:** chemotherapy, pineoblastoma, prognosis, radiation therapy

## Abstract

**Simple Summary:**

Pineoblastoma tumors are rare and aggressive tumors of the pineal gland. Due to their rarity, most of the literature on pineoblastoma consists of case reports and single-institution series. To better understand patient and clinical characteristics that influence survival in pineoblastoma patients, a systematic review and individual patient data meta-analysis were conducted. Another goal of this study was to determine whether or not patient outcomes had changed since the last systematic review on this topic was published in 2012. Patient survival was analyzed based on factors such as age, metastatic presentation, extent of surgical resection, adjuvant therapy, and publication year. Our study demonstrates that less-than-gross total resection, metastatic presentation, adjuvant chemotherapy without radiation, and an age of less than three years are associated with poorer survival. Furthermore, we found that the 5-year overall survival improved from 32.8% to 56.1% since 2012.

**Abstract:**

Most of the literature on pineoblastoma consists of case reports and single-institution series. The goal of this systematic review and individual patient data (IPD) analysis was to summarize the existing literature, identify factors associated with overall survival (OS), and provide a contemporary update on prognosis for patients with pineoblastoma. Forty-four studies were identified with 298 patients having IPD. Kaplan–Meier analyses were used to report survival outcomes based on age, tumor metastases, extent of resection (EOR), adjuvant therapy, and publication year. Cox regression was performed to identify independent predictors of time to mortality. Multivariable recursive partitioning analysis was used to identify the most important subgroups associated with mortality. Patients were classified based on publication year before and after the last systematic review on this topic (pre-2012 and 2012 onwards) and compared using univariate and multivariable analyses. This study demonstrates that EOR less-than-gross total resection, metastatic presentation, adjuvant chemotherapy without radiation, and tumor presentation in children less than three years old are associated with poorer prognosis. Since 2012, the 5-year actuarial OS has improved from 32.8% to 56.1%, which remained significant even after accounting for EOR, age, and adjuvant therapy. Pineoblastoma remains a severe rare disease, but survival outcomes are improving.

## 1. Introduction

Pineoblastoma (PB) is a rare and aggressive tumor of the pineal parenchyma that comprises less than 0.1% of intracranial neoplasms and is more common in children [1,2]. Pineal parenchymal tumors were historically classified as pineocytoma, pineocytoma-pineoblastoma, or pineoblastoma. In 2007, the World Health Organization further classified pineal parenchymal tumors as pineocytoma (Grade 1), pineal parenchymal tumors of intermediate differentiation (Grade 2 or 3), papillary tumors of the pineal region (Grade 2 or 3), and pineoblastoma (Grade 4) [3]. This grading scheme was unchanged in the World Health Organization’s 2021 update [4].

Although early studies supported conservative management with biopsy or subtotal resection (STR) to minimize morbidity [5,6], advances in microsurgical techniques and approaches for the pineal region have enhanced the standard of care with the option of more aggressive surgical resection with adjuvant radiation, chemotherapy, or both [7,8,9,10]. Nevertheless, approaches for pineoblastoma management remain heterogenous. Despite reports of the benefits of multimodality treatment [8,11], the use of adjuvant radiation ranges from 27 to 94% [12,13,14,15].

The last systematic review analyzing prognosis in pineoblastoma was published more than ten years ago and reported an overall survival (OS) of 54% at a mean follow-up of 31 months [11]. Two more recent analyses of national databases reported 5-year OS rates of 66% and 61%, respectively [14,16], suggesting that the prognosis may be improving. However, due to the rarity of this tumor, most of the available literature on pineoblastoma consists of case reports and single-institution case series with relatively low patient numbers that lack statistical power. The goal of this systematic review and individual patient data (IPD) analysis is to collect and reanalyze individual patient-level data in the literature [17], to summarize the existing literature, identify factors associated with OS, and provide an update on contemporary prognosis in patients with PB.

## 2. Methods

### 2.1. Design and Search

This study was performed per the Preferred Reporting Items for Systematic Reviews and Meta-Analyses (PRISMA) 2020 and PRISMA-IPD guidelines, and registered in the International Prospective Register of Systematic Reviews (PROSPERO, registration ID CRD42023424651) [17,18,19]. In November 2022, the PubMed (National Library of Medicine), Scopus (Elsevier), and Embase (Elsevier) databases were queried using the following search algorithms: “pinealoma” OR “pineocytoma” OR “pineoblastoma” OR “pineal parenchymal tumor of intermediate differentiation” OR “PPTID”) AND (“radiation” OR “radiosurgery” OR “gamma knife” OR “resection” OR “surgery”. No restrictions on date, article type, or language were applied as part of the search algorithm.

After aggregating the search results in Rayyan (Rayyan Systems Inc.) [20], duplicates were manually identified and removed. Two reviewers (K.R.N. and N.S.S.) independently screened articles by title and abstract for relevance and then screened remaining articles through a full-text review based on prespecified inclusion and exclusion criteria. The inclusion criteria included (1) articles published in English, (2) patients with histologically confirmed pineoblastomas, (3) those reporting treatment methods, (4) those reporting IPD, and (5) those for which the full text was available. The exclusion criteria included (1) conference abstracts, (2) existing reviews/meta-analyses without additional IPD, (3) nonhuman studies, (4) and studies with an overlap of patients from another study. Disagreements between reviewers were resolved by a tie-breaking reviewer (E.J.H.). Corresponding authors of twelve eligible studies that did not report IPD were contacted via email to request IPD, and additional de-identified IPD was received from one author.

### 2.2. Data Extraction

Study-level data extracted included the first author, title, year of publication, country of origin (defined based on the corresponding author’s address), study design, number of pineoblastoma patients with IPD, patient sex, and median patient age and follow-up time. The Grading of Recommendations Assessment, Development, and Evaluation framework was used to assess quality [21]. The Risk of Bias of Nonrandomized Studies of Interventions tool was used to determine the risk of bias [22]. The risk of bias for this study was determined based on the risk of bias of all included studies.

The IPD extracted included age, sex, presence of tumor metastasis, extent of resection (EOR), adjuvant radiation therapy (RT), total dose of radiation if applicable, adjuvant chemotherapy, and follow-up time. The primary outcome of this study was overall survival (OS). Metastasis was defined as a radiographic dissemination of pineoblastoma within the central nervous system, and the presence of tumor metastasis was only recorded if the publication explicitly mentioned evaluation for metastases. RT was classified as external beam radiation therapy (EBRT), stereotactic radiosurgery (SRS), or brachytherapy. The EOR was classified as none, biopsy only, subtotal resection, or gross total resection (GTR). Author descriptions of “near-total” resection were considered subtotal.

### 2.3. Statistical Analysis

Kaplan–Meier (KM) time-to-event analyses were used to report survival outcomes based on age, tumor metastases, EOR, adjuvant therapy, and publication year. Multivariable recursive partitioning analysis (RPA) was used to identify the most important subgroups associated with mortality. 

To evaluate changes in pineoblastoma prognosis since the publication of the last systematic review on this topic in 2012 [11], patients were also compared based on publication year before 2012 or 2012 onwards. For categorical data, Fisher’s exact tests were reported. The unpaired Wilcoxon rank sum test and Kruskal–Wallis test was used to compare medians between subgroups. The decision to use medians rather than means was based on statistically significant Shapiro–Wilks tests suggesting a non-normal distribution of each variable in at least one group of interest. Cox proportional hazards regression was performed to identify independent associations between age, EOR, sex, publication year (dichotomized as pre-2012 and 2012 onward), and adjuvant therapy, with time to mortality as the outcome.

Statistical analysis was performed using RStudio version 4.2.0, GraphPad Prism (Dotamatics) version 9.4.0, and JMP version 16 (SAS Institute Inc., Cary, NC, USA). Significance was defined as a *p*-value of <0.05. For multivariable analyses, only patients with available data for all variables of interest were included.

## 3. Results

### 3.1. Included Studies

**Figure 1:**
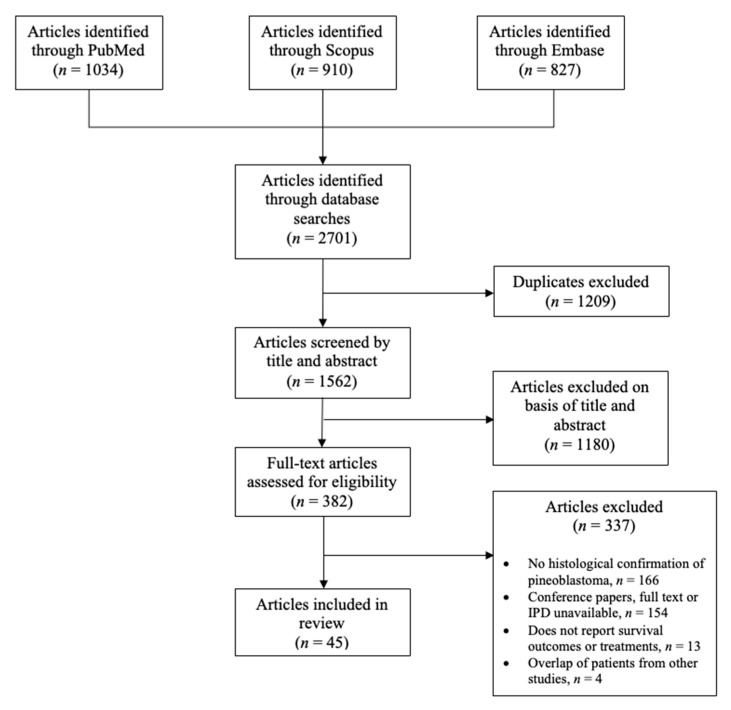
Preferred Reporting Systems for Systematic Reviews and Meta-Analyses flowchart.

Of the 2701 articles retrieved in the search, 44 studies with IPD on 298 patients with pineoblastoma were included in the systematic review (Table 1) [5,6,8,9,10,11,23,24,25,26,27,28,29,30,31,32,33,34,35,36,37,38,39,40,41,42,43,44,45,46,47,48,49,50,51,52,53,54,55,56,57,58,59,60,61]. Figure 1 presents the PRISMA flowchart for this systematic review [18]. Studies included both pediatric and adult patients. Briefly, 37 (84.1%) studies were case series and 7 (15.9%) were case reports. The most common country of origin was the United States (45.5%), followed by China, Germany, and Italy (6.8% each). The quality of most included studies was low. The risk of bias of most studies was high, predisposing this systematic review to a high risk of bias overall.

### 3.2. Individual Patient Data

The median (range) patient age was 11.7 (0.13–81.0) years. Forty-seven percent of patients were female, and 62.4% were pediatric (<18 years old). The median follow-up time for 214 patients with individually reported follow-up times was 27.6 (0.2–288.0) months, driven largely by mortality. Of the 226 patients assessed for metastases, 65 (28.8%) were metastatic at the time of treatment. Of the 256 patients with available information on the extent of resection, 11 (4.3%) underwent no surgical resection and were diagnosed at the autopsy, 52 (20.3%) underwent a biopsy alone, 127 (49.2%) received STR, and 66 (25.8%) received GTR. Adjuvant RT was reported in 274 (91.9%) patients. Of these, 205 (77.7%) received some form of external beam radiation therapy (EBRT), three (1.1%) received stereotactic radiosurgery (SRS), three (1.1%) received brachytherapy, and RT type was not reported in 55 (20.1%). Of the 240 patients in studies mentioning chemotherapy, 201 (83.8%) patients received chemotherapy (details of chemotherapy are reported in Table 1). Clinical and patient characteristics are summarized in Table 2.

### 3.3. Factors Affecting Patient Survival

**Figure 2:**
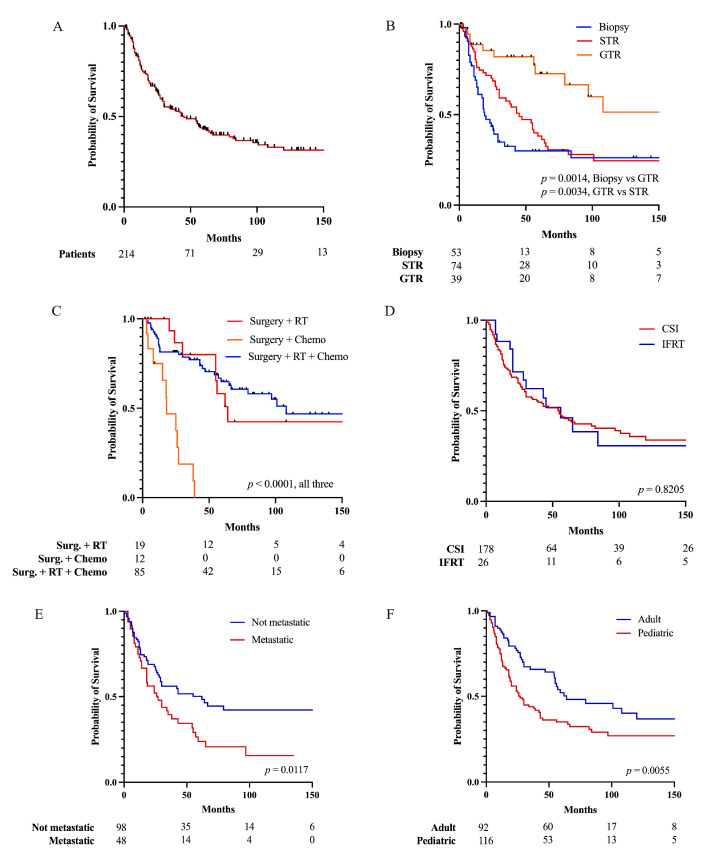
Kaplan–Meier analyses for (**A**) overall survival in pineoblastoma and comparison of patients between (**B**) extent of resection, (**C**) adjuvant treatment modality, (**D**) type of RT, (**E**) metastatic vs. non-metastatic presentation, and (**F**) adult and pediatric age at treatment.

In the entire cohort, 5-year OS was 43.1% (Figure 2A). Upon Kaplan–Meier analysis, a greater EOR resulted in longer 5-year OS (Figure 2B). Five-year OS was 72.6% in the GTR subgroup, compared to that of 38.2% in the STR subgroup (*p* = 0.0034) and 30.0% in the biopsy/no resection subgroup (*p* = 0.0014). Surgery with adjuvant RT and surgery with RT and chemotherapy were associated with a greater 5-year OS of 58.2% and 68.7%, respectively, compared to surgery with chemotherapy alone, which had no survivors at five years (*p* < 0.0001, Figure 2C). There was no difference in survival between patients receiving craniospinal irradiation and involved field radiation (*p* = 0.8502, Figure 2D). Patients whose tumors were not metastatic at treatment had a 5-year OS of 50.2%, compared to that of 23.8% (*p* = 0.0117) for patients with metastatic tumors (Figure 2E). Adult patients at the time of treatment had a 5-year OS of 52.1% compared to that of 35.0% for pediatric patients (*p* = 0.0055, Figure 2F). One, two, and five-year OS according to EOR, adjuvant therapy, metastases, and age are reported in Table 3.

**Figure 3:**
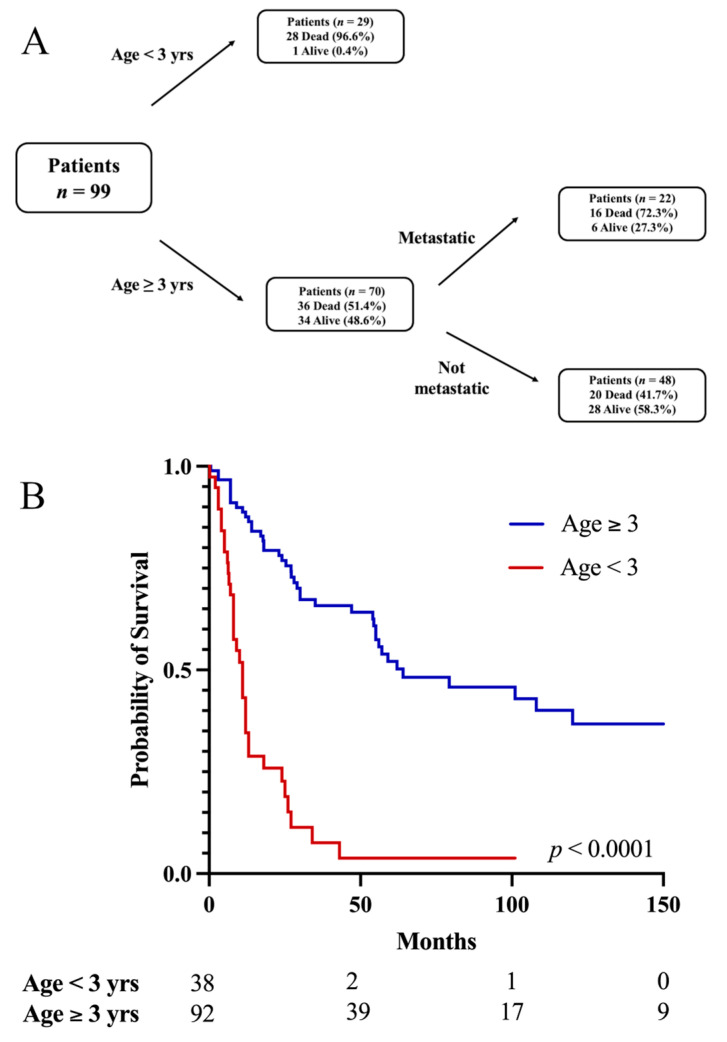
(**A**) Recursive partitioning analysis controlling for age, metastases, extent of resection, adjuvant RT, and chemotherapy optimally splitting mortality at age of three years. Patients younger than three years have greatest frequency of mortality at last follow up. Patients older than three years can be further grouped based on metastases at presentation, with patients with metastatic pineoblastoma having greater frequency of mortality at last follow up. (**B**) Age less than three years associated with lower 5-year OS.

Multivariable RPA accounting for age, metastases, EOR, adjuvant RT, and chemotherapy optimally split mortality at the age of three years (Figure 3A). Briefly, 97% of patients less than three years old were dead at the last follow up, compared to the 51.4% of patients older than three years old who were dead at the last follow up. Among the older patients, metastases were further predictive of mortality, with 72.3% of patients with metastases dead at the last follow up compared to the 41.7% of patients without metastases who were dead at the last follow up. Upon KM analysis, (Figure 3B), the 5-year OS for patients less than three years old was 3.8% compared to that of 52.1% for patients older than three years (*p* < 0.0001). Notably, patients who received adjuvant chemotherapy alone were younger (median age, 1.9 years (range 0.3–33)) than patients who received adjuvant RT alone (28.5 (1.3–70) years, *p* < 0.0001) or adjuvant RT and chemotherapy (12 (0.1–59) years, *p* < 0.0001).

### 3.4. Pineoblastoma Prognosis since 2012

**Figure 4:**
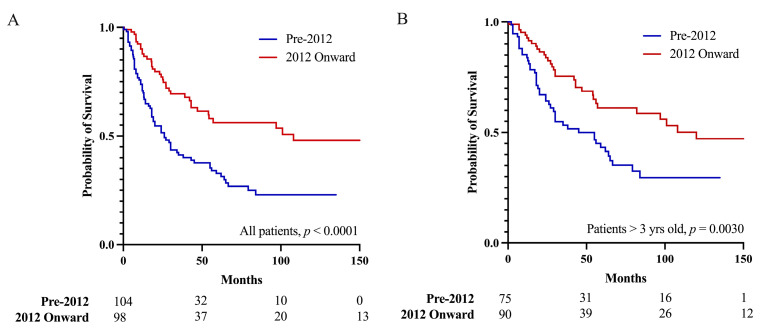
Publication year of 2012 onwards was associated with a better prognosis than publication before 2012 was for (**A**) all pineoblastoma patients, and (**B**) pineoblastoma patients older than three years, suggesting the prognosis of pineoblastoma is improving.

Patients in studies published 2012 onwards had a better 5-year OS than did patients in studies published before 2012 (56.1% vs. 32.8%, *p* < 0.0001, Figure 4A). When analyzing only patients older than three years, patients in studies published in 2012 onwards still had a better 5-year OS than did patients in studies published before 2012 (61.1% vs. 43.3%, *p* = 0.0030, Figure 4B). Upon univariate analyses (Table 2), patients in studies published in 2012 onwards were more likely to be female (52.9% vs. 39.3%, *p* = 0.0276), to be older (median age 16.0 vs. 8.6 years, *p* = 0.0012), to receive GTR (37.8% vs. 12.4%, *p* < 0.0001), and to receive lower doses of adjuvant radiation (56.5 vs. 88.0 Gy, *p* = 0.0019).

Upon multivariable Cox regression analyses (Table 4), an EOR less than GTR (HR = 2.0 (95% CI: 1.1–3.9), *p* = 0.0265), publication year prior to 2012 (HR = 1.7 (1.1–2.7), *p* = 0.0286), and age (HR = 0.99 (0.97–0.99) per year older, *p* = 0.0460) were independently associated with death after correcting for the effects of sex and adjuvant therapy.

## 4. Discussion

We present a systematic review and IPD analysis of prognostic factors in patients with PB. We demonstrate that an EOR less than the GTR, metastatic presentation, adjuvant chemotherapy without RT, and tumor presentation in children are significantly associated with poorer prognosis. More specifically, an age of less than three years is strongly associated with an increased hazard of death. Nevertheless, 5-year OS has improved since 2012, even when controlling for EOR, age, and adjuvant therapy.

A younger age at diagnosis, metastatic presentation, and less-than-gross total resection have previously been recognized as risk factors for worse prognosis [11,36,62]. The anatomy of the pineal region presents technical challenges for the surgeon [63], which is why more conservative approaches dominate the early literature on the management of pineoblastomas [5,6]. Nevertheless, in agreement with previous studies [11,14], our analysis demonstrates that GTR, followed by adjuvant RT or a combination of RT and chemotherapy yields the best prognosis. Although several chemotherapy regimens for pineoblastoma have been reported [9,26,29,36,41,42,47,57], there is currently no convincing data on chemotherapy alone as an adjuvant treatment modality in PB. In the current study, patients receiving chemotherapy alone after surgery had very poor outcomes compared to patients receiving adjuvant radiation with or without chemotherapy. This suggests that RT may be an essential adjuvant modality, but since patients receiving chemotherapy alone after surgery were significantly younger, selection bias may heavily influence these outcomes. In addition, there appears to be an additive benefit of a triple-modality approach (surgery + RT + chemotherapy) compared to a dual-modality (surgery + RT) approach (Figure 2C). Considering the rarity of the tumor and the historically poor outcomes, trials utilizing historical controls and/or radiographic endpoints may be considerations when evaluating novel therapeutic approaches. 

In their earlier systematic review, Tate et al. identified an age of younger than five years as predictive of poorer prognosis [11]. Our updated analyses, however, demonstrate that the age cutoff may in fact be younger, at three years. Recent studies by Mynarek et al., and Handsford et al. similarly found an age of three years to be critically predictive of worse prognosis [7,64]. A 2017 study by Raleigh et al. in very young pediatric embryonal brain tumor patients found that post-surgical RT delivered before disease recurrence resulted in better outcomes than did RT at the time of recurrence [65]. Thus, it is possible that the poor outcomes are driven, at least in part, by the omission or delay of RT in this patient population due to concern for its deleterious toxicities in the very young.

The importance of age in pineoblastoma prognosis likely reflects different underlying molecular mechanisms that drive the aggressive behavior of pineoblastoma in younger children. Indeed, recent studies show that molecular heterogeneity plays a large role in determining prognosis in pineoblastoma, and molecular subgroups have been defined by the 2021 WHO grading. A 2020 clinicopathologic study by Li et al. found that pineoblastomas could be stratified into three subgroups based on molecular features. The most aggressive subgroup, defined by alterations of the MYC/RB axis, was found much more commonly in the youngest patients [66]. Another study by Liu et al. used genome-wide DNA methylation profiling and next-generation sequencing to identify pineoblastoma subgroups with tissue from 43 patients. The most common subgroup, PB-B, was distinguished by alternations in the miRNA-processing pathway genes *DICER1*, *DROSHA*, and *DGCR8* and associated with an older age at diagnosis and longer PFS [67], reinforcing and elaborating upon previous studies establishing *DICER1* alterations as important drivers in pineoblastoma [68,69]. In fact, the absence of *DICER1* and *DROSHA* mutations differentiates PPTID from pineoblastoma [70]. Liu et al. also discovered two novel subgroups, PB-B-like and PB-FOXR2. Compared to PB-B tumors, PB-B-like tumors occurred in older patients with localized disease with more favorable outcomes [67]. The clinically aggressive PB-FOXR2 subgroup had a universal overexpression of *FOXR2*, a proto-oncogene that promotes the transcription of MYC to enhance cellular proliferation [67]. In the context of these recent discoveries, the World Health Organization’s 2021 classification for pineoblastoma defined four molecular subtypes: miRNA processing altered 1, miRNA processing altered 2, RB1-altered, and MYC/FOXR2-activated, each with a distinct prognosis [4]. This molecular subgrouping has high prognostic value [66,67,71]. Depending on the molecular subtype, 5-year OS can range from 68% and 100% for miRNA processing altered 1 and miRNA processing altered 2 subgroups, respectively to 38% and 23% for the RB1-altered and MYC/FOXR2-activated subgroups [66]. Moving forward, it will be interesting to see how the clinical variables associated with outcomes that we report in this meta-analysis correlate with the molecular subgroups.

Our analysis also demonstrated an improvement in outcomes since 2012. This improvement is likely driven at least in part by an increase in the proportion of pineoblastoma patients receiving GTR, which was also independently associated with time to mortality. There were also increases in the number of female pineoblastoma patients and in the median age of patients, both factors which have been associated with a better prognosis [11,14]. However, only the publication of results before 2012, an EOR less than the GTR, and age were independently associated with worse outcomes upon multivariable modeling. Although age was a significant predictor, the effect size was very small (HR = 0.99 per year older), suggesting that improvements reflected in the literature are not due to demographic changes alone, and again emphasizing the importance of achieving GTR whenever possible. Future studies including molecular subgrouping are necessary to determine whether this improvement in survival is related to improvements in surgical techniques, or simply reflects differences in tumor biology in the different populations.

### Limitations

This study was at a high risk of bias because all included studies were retrospective case series or case reports, limiting the extrapolation of results. The proportion of adult patients in our cohort was greater than that seen in practice [72], which might bias our estimates of OS. Furthermore, there was heterogeneity in the variables reported across studies, and even among individual patients within studies. To maximize statistical power and minimize bias, we included the maximum number of patients possible for each analysis. It is possible that the inclusion of complete data from all patients would yield different magnitudes of effect. Additionally, we were restricted to variables that are commonly reported in the literature. It is possible that the inclusion of variables such as surgical approach would have provided greater clarity to clinicians on optimizing treatment in patients with pineoblastoma. Furthermore, patient stratification according to tumor molecular characteristics may show that optimal treatment strategies differ by molecular subgroup, but due to the lack of data this remains to be seen. As we sought to include tumors classified by the World Health Organization in the initial screening for this review, relatively rare entities, such as pineal anlage tumors [73,74], were not included. It is also possible that with contemporary molecular neuropathologic techniques, some of the patients included in the current study, especially older adults reported in studies prior to 2007, would have had their tumors reclassified [10]. Lastly, there were some studies that did not report disaggregated data and did not respond to requests for de-identified IPD, which reduced the number of patients available for IPD analysis.

## 5. Conclusions

Pineoblastoma continues to have a poor prognosis, but OS has increased significantly in the past decade. Tumor metastases, an EOR less than the GTR, adjuvant chemotherapy without radiation, and an age of less than three years are significantly associated with poorer prognosis. These data stress the importance of GTR followed by adjuvant RT in maximizing OS.

## Figures and Tables

**Table 1:** Studies included in systematic review of patients with pineoblastoma.

Author	Country	Study Design	Num. of PB Patients	Quality ^†^	Risk of Bias ^†^	Female, *n*	Age (yrs) *	Follow-Up (Month) *	Chemotherapy
Herrick et al., 1979 [46]	USA	Case series	10	Low	Serious	4 (40%)	9.5 (4.6–39)	32 (21.4–74.9)	
Borit et al., 1980 [6]	UK	Case series	7	Low	High	2 (29%)	18 (5.5–52)	18 (3–84)	
Jooma et al., 1983 [5]	UK	Case series	3	Low	Serious	2 (67%)	11 (1.3–23)	18 (6–24)	
Lesnick et al., 1985 [52]	USA	Case series	2	Very low	Serious	2 (100%)	27.5 (12–43)	26	Lomustine
Linggood et al., 1992 [53]	USA	Case series	4	Low	High	2 (50%)	18.5 (3–35)	23.5 (16–72)	
Kellie et al., 1992 [50]	USA	Case series	1	Very low	Serious	1 (100%)	13	28	Carboplatin and etoposide
Ghim et al., 1993 [41]	USA	Case series	3	Low	High	Not reported	5 (3–7)	24 (5–60)	Cisplatin, etoposide, vincristine
Chang et al., 1995 [31]	USA	Case series	9	Low	High	3 (33%)	35 (17–59)	26 (6–62)	6-Thioguanine, dibromodulcitol, lomustine, procarbazine, vincristine
Duffner et al., 1995 [36]	USA	Case series	11	Low	High	3 (27%)	0.67 (0.13–3)	11 (3–34)	Cisplatinum, cyclophosphamide, etoposide, vincristine
Jakacki et al., 1995 [9]	USA	Case series	25	Low	High	Not reported	3.1 (1.5–19.6)	Not reported	Lomustine, prednisone, vincristine OR Cisplatin, cytarabine, cyclophosphamide, hydroxurea, lomustine, methylprednisone, procarbazine
Ashley et al., 1996 [26]	USA	Case series	8	Low	High	2 (25%)	21 (3–23)	70.5 (19–131)	Cyclophosphamide
Prados et al., 1999 [58]	USA	Case series	5	Low	High	1 (20%)	28 (26–30)	55 (14–64)	
Charafe-Jauffret, et al., 2001 [32]	France	Case series	2	Low	Serious	0	22 (18–26)	68.5 (29–108)	
Hasegawa et al., 2002 [45]	USA	Case series	3	Low	High	1 (33%)	43 (15–61)	17 (7–56)	
Broniscer et al., 2004 [29]	USA	Case series	7	Low	High	5 (71%)	1 (0.9–7.2)	12 (0.2–101)	Etoposide, thiotepa ± carboplatin
Cuccia et al., 2006 [35]	Argentina	Case series	12	Low	High	4 (25%)	6.6 (0.5–18.1)	9.6 (6.3–79.3)	Cisplatin, lomustine, vincristine
Hinkes et al., 2007 [47]	Germany	Case series	11	Low	High	4 (36%)	3.6 (0.6–16.9)	26 (4–130)	Cisplatin, lomustine, vincristine
Gilheeney et al., 2008 [42]	USA	Case series	11	Low	High	5 (45%)	8.3 (0.25–13.6)	61 (3–135)	
Tate et al., 2009 [59]	USA	Case report	1	Very low	Serious	1 (100%)	18	24	Cisplatin, lomustine, vincristine
Maarouf et al., 2010 [54]	Germany	Case series	7	Low	High	1 (17%)	36 (6–65)	Not reported	
Nozza et al., 2010 [56]	Italy	Case report	1	Very low	Serious	0	0.75	9	
Panosyan et al., 2011 [57]	USA	Case series	1	Low	High	1 (100%)	10	Not reported	AHSCR **, temozolomide
Ito et al., 2014 [48]	Japan	Case series	3	Low	High	1 (33%)	36 (30–49)	61 (45–122)	Cisplatinum, etoposide, ifosfamide
Farnia et al., 2014 [8]	USA	Case series	31	Low	High	21 (68%)	18 (0.25–52)	38.4 (0.2–332.4)	
Raleigh et al., 2014 [10]	USA	Case series	29	Low	High	14 (48%)	19 (1–57)	49.2 (1–275)	Multiple different regimens
Friedrich et al., 2014 [38]	Germany	Case series	10	Low	High	4 (40%)	30.5 (22–57)	32 (21.4–59.5)	Cisplatin, lomustine, vincristine or SKK, methotrexate/leucovorin
Ai et al., 2015 [24]	China	Case report	1	Very low	Serious	0	46	36	
Golbin et al., 2015 [43]	Russia	Case report	1	Very low	Serious	1 (100%)	23	57	Cisplatin, cyclophosphamide, etoposide
Gener et al., 2015 [40]	USA	Case series	12	Low	High	10 (83%)	44.5 (24–81))	55 (0.5–288)	Vincristine
Alsultan et al., 2015 [25]	Saudi Arabia	Case series	1	Low	High	0	3	13	Cisplatin, cyclophosphamide, etoposide, vincristine
Biswas et al., 2015 [28]	India	Case series	17	Low	High	6 (35%)	14 (4–47)	30.3 (1.9–69.3)	Carboplatin, etoposide OR Carboplatin, vincristine, and etoposide
Kumar et al., 2018 [51]	India	Case series	2	Low	Serious	0	12.5 (12–13)	31.5 (20–43)	Cisplatin, etoposide
Horiba et al., 2018 [61]	Japan	Case report	1	Very low	Serious	Not reported	0.25	7	
Kang et al., 2018 [49]	Taiwan	Case series	11	Low	High	6 (55%)	2.8 (1.5–16.8)	27 (8–190)	
Tian et al., 2018 [60]	China	Case series	18	Low	High	10 (56%)	2.8 (1.6–13)	Not reported	Doxorubicin, vincristine, ifosfamide
Abbassy et al., 2018 [23]	Egypt	Case series	2	Low	High	0	3 (2–4)	22.6 (12–33)	
Choque-Velasquez et al., 2019 [33]	Finland	Case series	3	Low	High	3 (100%)	18 (3–25)	170 (152–173)	
Cuccia et al., 2019 [34]	Italy	Case report	1	Very low	Serious	1 (100%)	21	12	Cisplatin, lomustine, vincristine
Elshahoubi et al., 2019 [37]	Jordan	Case series	1	Low	Serious	1 (100%)	33	17.8	
Gaito et al., 2019 [39]	Italy	Case report	1	Very low	Serious	0	46	36	Cisplatin and etoposide
Bernstock et al., 2020 [27]	USA	Case series	1	Very low	Serious	Not reported	3.1	95	Cisplatin, cyclophosphamide, etoposide, high-dose methotrexate, vincristine
Görgün et al., 2021 [44]	Turkey	Case series	6	Low	High	3 (60%)	5.8 (2–14)	66 (12–228)	Cyclophosphamide, etoposide, vincristine OR carboplatin, etoposide, ifosfamide, OR Cyclophsophamide, lomustine, procarbazine, vincristine
Nguyen et al., 2021 [55]	USA	Case report	1	Very low	Serious	0	22	120	Vincristine
Cai et al., 2021 [30]	China	Case series	1	Low	High	0	5	30	

* Age and follow up are reported as medians (ranges) and calculated for patients included in the IPD analysis. ** AHSCR, autologous hematopoietic stem cell rescue. ^†^ Study quality was determined using the Grading of Recommendations Assessment, Development, and Evaluation framework. Risk of bias was determined using the Risk of Bias of Nonrandomized Studies of Interventions tool.

**Table 2:** Clinical and patient characteristics.

Variable	Value/Pts. with Data	Pre-2012	2012 Onward	*p*-Value
Total Patients	298	144	154	
Female	127/270 (47.0%)	46/117 (39.3%)	81/153 (52.9%)	0.0276
Follow up, median (range)	27.1 (0.2–288.0) mo			
Age, median (range)	11.7 (0.13–81.0) yrs	8.55 (0.13–65.0)	16.0 (0.25–81.0)	0.0012
Pediatric	186/298 (62.4%)			
Metastatic at treatment	65/226 (28.8%)	40/126 (31.7%)	25/99 (25.3%)	0.3033
Extent of resection				<0.0001
None	11/256 (4.3%)	6/121 (5.0%)	5/135 (3.7%)	
Biopsy	52/256 (20.3%)	28/121 (23.1%)	24/135 (17.8%)	
Subtotal	127/256 (49.2%)	72/121 (59.5%)	55/135 (40.7%)	
Gross total	66/256 (25.8%)	15/121 (12.4%)	51/135 (37.8%)	
Adjuvant RT	274/298 (91.9%)	107/132 (81.1%)	116/142 (81.7%)	0.3373
EBRT	213/274 (77.7%)	105/132 (79.5%)	108/142 (75.8%)	
SRS	3/274 (1.1%)	1/132 (0.01%)	2/142 (1.4%)	
Brachytherapy	3/274 (1.1%)	3/132 (2.3%)	0	
Not defined	55/274 (20.1%)	35/132 (26.5%)	22/142 (16.7%)	
Adjuvant RT dose, Gy	80.1 (38.0–181.4)	88.0 (38.0–181.4)	56.5 (40.0–158.0)	0.0019
Chemotherapy	201/240 (83.8%)	22/125 (17.6%)	17/115 (14.8%)	0.6021

**Table 3:** One, two, and five-year overall survivals for different subgroups.

Variable	1-Year OS	2-Year OS	5-Year OS
Overall	78.5%	64.2%	43.1%
EOR			
Biopsy/no resection	69.1%	43.4%	30.0%
GTR	88.7%	85.4%	72.6%
STR	80.4%	70.1%	38.2%
Adjuvant therapy			
Chemo	75.0%	46.9%	0%
RT	100%	86.7%	58.2%
CSI	78.3%	65.3%	45.7%
IFRT	88.3%	71.5%	46.1%
RT and chemo	85.3%	81.5%	64.9%
Metastases			
No	79.0%	67.7%	50.2%
Yes	72.9%	52.1%	23.8%
Age			
≥18 years old	87.6%	76.9%	52.1%
<18 years old	71.1%	53.1%	35.0%
≥3 years old	87.6%	76.9%	52.1%
<3 years old	34.6%	22.7%	3.8%
Publication year			
Pre-2012	70.8%	51.6%	56.1%
2012 Onward	87.8%	77.2%	32.8%

**Table 4:** Results of cox proportional hazards analysis of time to mortality.

Variable	HR	HR 95% CI	*p*-Value
STR/Biopsy/None *	2.0	1.1–3.9	0.0265
Female sex	1.4	0.9–2.2	0.1496
Age per year older	0.99	0.97–0.99	0.0460
Surgery + Chemo **	1.3	0.7–2.2	0.4691
Publication year pre-2012	1.7	1.1–2.7	0.0286

* Relative to GTR; ** relative to surgery with adjuvant RT or surgery with adjuvant chemo and RT.

## Data Availability

This systematic review was based on previously published data which can be accessed from the primary sources.
